# Public Perceptions, More Than Misinformation, Explain Poor Adherence to Proven COVID-19 Control Measures

**DOI:** 10.4269/ajtmh.21-1251

**Published:** 2022-02-09

**Authors:** Bernard Seytre

## Abstract

Since the beginning of the COVID-19 pandemic, there has been a profusion of studies and webinars on the infodemic (the rapid diffusion of information on the internet). The infodemic is often cited as a key factor in the lack of adherence to COVID-19 preventive measures, including vaccination. A study we conducted in West Africa questions the reality of this impact: the majority of people who do not adhere to the preventive measures draw their opinion from their own experience, not from what they have viewed or read on social networks. Historically, resistance to public health messages and interventions, including vaccination, existed before the advent of the Internet. Studying the perceptions of the population and not only the circulation of information is necessary to fully understand the lack of adherence to the COVID-19 preventive measures and to build an effective communication strategy.

## INTRODUCTION

The declaration from WHO Director-General Tedros Adhanom Ghebreyesus on February 15, 2020, that “We are not just fighting an epidemic, we are fighting an infodemic,” opened the floodgates to a profusion of studies and webinars on the COVID-19 infodemic. The infodemic is defined as “an abundance of information, right or wrong, that accompanies an epidemic,” ^1^ but Dr. Ghebreyesus only mentioned the “fake news” that *“*spreads faster and more easily than this virus, and is just as dangerous.” COVID-19 infodemic studies confirmed a point already demonstrated: a rumor is three times more likely to be shared than verified information. ^2^ But are individuals three times more likely to believe rumors rather than accurate information? The impact of an infodemic on knowledge, beliefs, and hopes should not be assumed. It should be studied. A recent British Royal Society comprehensive report concluded: “Although misinformation content is prevalent online, the extent of its impact is questionable.” ^3^

Authors have argued that “the adverse effects of the infodemic may be exacerbated in low- and middle-income countries (LMICs),” implying that Internet users are more likely to believe digital nonsense in Ouagadougou, Quito, or Kuala Lumpur than in Paris, Geneva, or New York. ^4^ However, the results of a mixed socioanthropological study that we conducted in five LMICs in West Africa (Burkina Faso, Ivory Coast, Sierra Leone, Guinea-Bissau, Cabo Verde) have led us to question the reality of this impact attributed to the infodemic.

As an example, our quantitative survey showed that although the take-up of face masks in places such as public transport was low in the countries we surveyed, the population had received the messages promoting the wearing of masks. Furthermore, the vast majority believed that wearing a mask was a useful recommendation to protect against COVID-19, with levels ranging from 75.7% to 98.3%, depending on the country. ^5^ Therefore, it was not erroneous ideas circulating about the preventive measures that lead to the lack of adherence, but rather the idea that it was pointless to apply the measures. We showed that only 20.1% of the people surveyed knew that asymptomatic people can transmit the disease (3.2% to 39.7% according to the country), which implies that 79.9% believe they are at risk of infection only in the presence of a sick person.

In the qualitative portion of our study, we interviewed 24 individuals in Burkina Faso, Ivory Coast, and Sierra Leone who had expressed erroneous ideas about the pandemic, the majority of whom thought that COVID-19 was not present in their country. ^6^ This majority was aware that the disease exists elsewhere in the world, based on images they had seen of sick and dead patients in Europe, the United States, Brazil, and elsewhere, through television channels such as France 24 or the BBC, either live or via YouTube, yet they said they had never seen images of people affected with COVID-19 in their own country on national television. Respondents also stated that they did not personally know anyone around them who had contracted COVID-19. According to a study conducted from September to December 2020 in 15 African countries, only 1% to 8% of the respondents knew someone who had tested positive for the COVID-19 virus, and more than half thought that the threat from the coronavirus was exaggerated. ^7^ The only mention of social networks in support of the claims among the interviewees of our study was as platforms to watch the television news of the channels cited. Fake news and conspiracy theories were not mentioned.

Our study was conducted in five West Africa countries; however, its lessons can probably be extended. One of the rare studies on the impact of the infodemic on knowledge and beliefs around COVID-19, conducted in eight European, American, and Asian countries, showed that more than 90% of those interviewed did not believe any of five statements of misinformation on COVID-19 vaccines that were circulating on social networks and which were submitted to them in the study. ^8^ Another study found, among other results, that 7.4% of people in the United Kingdom, Ireland, the United States, Spain, and Mexico thought that breathing hot air could kill the virus and that 16% believed 5G wireless technology renders one more susceptible to the virus. ^9^ The authors noted “a clear link between susceptibility to misinformation and vaccine hesitancy” yet acknowledged that “causality can run both ways: being vaccine hesitant may, in turn, also lead people to become more susceptible to misinformation.” Are people refusing vaccination because of their belief in misinformation, or are they refusing vaccination for other reasons and consequently looking for arguments to support their decision? The French population is exposed to the same misinformation circulating on the Internet, yet 93% of French individuals aged 18 years and older have received one dose of a COVID-19 vaccine in the mainland departments of France, whereas this is the case for only 47% of the population in the French overseas department of Guadeloupe, where there have been massive protests and riots against COVID-19 vaccination and various rumors about the vaccines are widely circulated by the protestors, according to press reports. ^10^^–^ ^12^ Drivers of the protests appear to include feelings of social injustice and distrust of the faraway national government.

Our own fieldwork and the aforementioned studies reinforce conclusions published early in the pandemic, which showed that “personal experience with the virus” is crucial to the perception of the risk of COVID-19 and that the “risk perception of COVID-19 consistently correlates strongly with a number of experiential and sociocultural factors.” The authors emphasize that communication therefore must “involve much more than just getting the numbers right.” ^13^ These findings suggest that it is of greater importance to capture a better understanding of the perceptions among the population to determine the information that should be communicated, as well as how and by whom this information should be promoted, rather than simply counteracting false information with accurate facts.

Examples abound to illustrate that rumors and misunderstanding around vaccination campaigns did not emerge with the advent of social networks: during the French colonization of Algeria, the smallpox vaccine was rumored to be a tool of forced Christianization; in Cameroon in 1990, the tetanus vaccine was accused of sterilizing women; in France in the late 1990s, the hepatitis B vaccine was believed to cause multiple sclerosis; in the United Kingdom, the measles, mumps, and rubella vaccine has been accused of causing autism; and the oral vaccine against poliomyelitis has been accused of sterilizing children in African and Asian countries, to name a few examples. The Internet allows rapid and abundant access to information provided by governments, the media, scientists, and rumor mongers. In the past, such information was obtained through the radio, television, printed journals, and word of mouth. Although the Internet is undoubtedly an accelerator of the dissemination of both accurate and inaccurate information, people today, as in the past, tend to draw from this information those elements that correspond to their perceptions and, for some, their political agenda.

Is there any way to counter the spread of misinformation around COVID-19 circulating on the Internet? Despite the considerable number of publications on the COVID-19 infodemic, solutions have yet to be found. A systematic review of COVID-19–related misinformation on social media published in the *Bulletin of the World Health Organization* in March 2021 stated that “the most effective strategies for tackling COVID-19–related misinformation are currently not known. Although there are many ongoing attempts to correct misinformation, we were unable to identify any study that examined the effects of these attempts, such as whether they enabled people to be better informed or helped them feel safer.” ^14^ Although there is no direct way to counter misinformation, we can attempt to limit its impact by reinforcing the level of accurate knowledge among the population—in other words, elevating health literacy on COVID-19 to help people make better-informed decisions. ^15^ We could view this approach as building “informed consent” to the public health measures.

Studying the existing rumors can provide an indication of the information to which the population is exposed but does not fully capture what the population perceives and the correlating reasons for adherence or nonadherence to preventive measures, including vaccination. First, to obtain a complete picture of this information, studies should also take into consideration the accurate information transmitted through the Internet. Second, and more importantly, the emphasis on infodemic studies and management tends to reinforce vertical communication based on the views of the specialists rather than the perceptions of the population.

One cannot propose an intelligible epidemic control policy and design a relevant communication strategy without studying the perceptions of the population. Although considerable human and financial resources have been dedicated to the infodemic, its real impact on peoples’ adherence to COVID-19 prevention measures has not been established. It would be more useful to redirect some of these resources toward obtaining a clear picture of the COVID-19 health literacy of target populations through social sciences studies.

First, determining the COVID-19 health literacy level will allow communication campaigns to avoid inappropriate messages that could inadvertently raise doubts and distrust. For example, an overemphasis on the need to get vaccinated could raise suspicions among people who believe they are not at risk of being infected, opening the door to conspiracy theories around the government push on vaccination. Second, determining COVID-19 health literacy will allow communication campaigns to design more effective plans and messages. To take West Africa as an example, when a substantial number of people believe that COVID-19 is not present in their country or that they can only be infected by sick people, communication should focus on demonstrating the presence and transmission of the pandemic in the country by broadcasting television news images of sick people, for example, and on explaining asymptomatic transmission of the virus.

Changing the focus from the infodemic to the actual perceptions of the population is a necessary condition to build an “evidence-based” COVID-19 communication strategy in which the “evidence” includes the perceptions of the population.
